# Stigma associated with medication treatment for young adults with opioid use disorder: a case series

**DOI:** 10.1186/s13722-018-0116-2

**Published:** 2018-05-07

**Authors:** Scott E. Hadland, Tae Woo Park, Sarah M. Bagley

**Affiliations:** 1 Division of General Academic Pediatrics, Department of Pediatrics, Boston University School of Medicine, Boston Medical Center, Boston, MA USA; 2 Department of Psychiatry, Boston University School of Medicine, Boston Medical Center, Boston, MA USA; 3 Clinical Addiction Research and Education Unit, Section of General Internal Medicine, Department of Medicine, Boston University/School of Medicine, Boston Medical Center, Boston, MA USA; 4 Grayken Center for Addiction, Boston Medical Center, Boston, USA

**Keywords:** Stigma, Medication treatment, Young adults

## Abstract

**Background:**

Opioid-related overdose deaths have risen sharply among young adults. Despite this increase, access to evidence-based medication for opioid agonist treatment (OAT) for youth remains low. Among older adults, barriers to OAT include the paucity of buprenorphine-waivered prescribers and low rates of prescribing among waivered physicians. We have increasingly found in our clinical practice significant stigma related to using OAT to treat addiction for young adults. In this series, we describe three cases of young adults who faced significant stigma related to their treatment.

**Case presentations:**

The first case is a young male with a history of significant trauma and a severe opioid use disorder. He started buprenorphine and has found a job, stayed *abstinent*, and began a healthy relationship. At each step in his recovery, he has faced resistance to taking medication from other treatment providers, directors of *sober houses*, and his parents. The second case is a young woman who presented to a substance use treatment program after a relapse. She was unable to restart buprenorphine despite our calling to ask that it be restarted. Ultimately, she left against medical advice and was stabilized as an outpatient on buprenorphine. The final case is a young woman who stopped buprenorphine after being told she was “not sober” while attending 12-step group but restarted after conversations with her clinical team. In each case, the patient has continued their medication treatment and are stable.

**Conclusions:**

Opioid-related deaths continue to rise among all age groups, including young adults. Stigma related to medication treatment can be a substantial barrier for many young adult patients but there are concrete steps that providers and communities can take to address this stigma.

## Introduction

Opioid-related overdose deaths have risen sharply among 18–25 year olds (young adults). From 2014 to 2015, there was a 72% increase in young adult deaths related to synthetic opioids (including fentanyl) and 15% increase in heroin-related deaths [1]. There is consensus that opioid agonist treatment (OAT)—including use of buprenorphine, methadone, or naltrexone—should be offered to people of all ages with an opioid use disorder (OUD) [2]. This consensus is shared among federal and state authorities, public health experts, and professional medical societies.

Access to OAT for youth remains low [3]. A recent study showed that from 2000 to 2014, only 1 in 4 youth diagnosed with OUD received medication, and that treatment rates have largely plateaued since 2009 [4]. Given that medication treatment has been shown to improve outcomes including human immunodeficiency virus transmission, hepatitis C transmission, retention in treatment, and mortality, use of medications, when appropriate, should be highly encouraged [2, 5]. Common reasons cited for poor access to OAT in the adult population include the paucity of buprenorphine-waivered prescribers, low rates of prescribing among waivered physicians, and critically, stigma associated with both the disease of addiction and use of medications [5–8]. Although many of these barriers have been identified in the treatment of older adults, it is increasingly clear that youth face similar barriers [9]. As a result, strategies to expand access to medications have included increasing the number of prescribers and their prescribing rates, as well as decreasing stigma related to a substance use disorder and treatment.

We have found in our clinical practice that many youth experience stigma specifically related to receiving OAT. We work in a primary care-based addiction treatment program serving patients under age 25. Our team is multidisciplinary and includes social workers, nurses, recovery coaches, and physicians. In our experience, young adults are often hesitant to initiate OAT because trusted and often well-intentioned adults (including treatment providers, parents, and other caregivers) have recommended against OAT.

We received permission from three patients to share their experiences which have been representative of what we have heard from many others.

## Illustrative cases

#1 We first met MH, a male in his early 20s, in the fall of 2016. We diagnosed him with severe OUD and started buprenorphine. He was adherent with his early visits but the treatment team felt his underlying mental disorders which included depression and post-traumatic stress disorder (PTSD), would be a barrier to his recovery unless his co-occurring addiction and mental disorders were concurrently addressed. Despite our efforts to provide patient-centered care, his co-occurring mental disorders resulted in an inpatient hospitalization. He was discharged to a residential treatment program. At that point, he contacted us with a request to restart buprenorphine. Over the following few months, he engaged in psychotherapy including for PTSD, found a job, started a relationship with a supportive girlfriend, and has remained abstinent. However, he has repeatedly faced resistance in continuing buprenorphine treatment. At the residential treatment program, staff asked repeatedly about the need for medication and questioned whether he could really be abstinent while taking buprenorphine, despite his voiced desire to stay on the medication. When he transitioned to a sober house, the director questioned the dose of buprenorphine and shared concerns with his parents about being on buprenorphine. His parents also questioned the need for medication. To respond to the resistance to buprenorphine treatment, we saw this patient regularly and provided a consistent message that it is was clear that buprenorphine was helping him achieve his recovery goals. Although he has remained on buprenorphine, he has been clear with us that this resistance (from other caregivers?) to staying on buprenorphine has been difficult to manage.

#2 GA, a 21-year-old female with severe OUD, entered our program soon after having a baby. She had come to prenatal care late in her pregnancy and had been started on buprenorphine. After delivery, she was living at home with her baby and mother and received little recovery support. Despite the efforts of her mother and our team to engage her in recovery support, her worsening sense of isolation led to a relapse in drug use and we did not see her for about a month. Her mother eventually called to inform us that the patient had decided to go to a detoxification program and wanted to restart buprenorphine. We attempted to restart her buprenorphine in coordination with the detoxification program. Different members of our team (physician, nurse, social worker) were informed by the detoxification program staff that we were being manipulated by the patient. One staff person informed us that there was no reason to start buprenorphine since (1) the patient was no longer in active opioid withdrawal and (2) her most recent opioid use was “just a binge”. I (SMB) tried to explain that she was still having cravings and that cravings are an indication to begin treatment with buprenorphine. However, they refused to begin treatment. She eventually left detoxification against medical advice. We restarted buprenorphine and helped facilitate admission into a residential program. She has been abstinent since that time and has been stable on buprenorphine.

#3 LD, a 20-year-old female with severe OUD, bipolar I disorder, and PTSD presented to our program after having recently moved from across the country to attend college. She had previously been stable on buprenorphine in another treatment program prior to her move, but after starting college, had begun to use heroin again and wanted to reinitiate buprenorphine and reengage in treatment. We successfully performed an induction on buprenorphine/naltrexone, and subsequently, she ceased heroin use, corroborated by urine drug tests. As a component of her treatment, she also began attending a 12-step program to receive peer recovery support. One month into treatment, however, she presented in crisis, having reinitiated prescription opioid use, with a urine drug test positive for fentanyl (a common contaminant in counterfeit prescription opioid pills in Massachusetts) as well as benzodiazepines. After meeting with our team, it was clear that LD had suddenly stopped taking her buprenorphine/naltrexone, and had taken prescription pills she had purchased from a friend after experiencing severe cravings. LD revealed that at her 12-step program, she had received messaging that she was “not truly sober” while on buprenorphine/naltrexone, and so had suddenly attempted to stop her OAT. After a careful discussion with our clinical team regarding the potential benefits and risks of OAT, LD elected to reinitiate buprenorphine/naltrexone. She has continued to attend 12-step meetings, electing not to share with her peers that she is on OAT, and to date, has not used any other opioids and remains engaged in care.

## Discussion

Opioid-related deaths continue to rise among all age groups, including young adults. Medication treatment is a key response to address overdose deaths but there have been barriers expanding access to OAT. Stigma related to medication treatment can be a substantial barrier for many young adult patients.

There are several reasons why many patients, young or old, experience stigma when accessing OAT. Historically, most addiction treatment has occurred separate from primary care and for many years, there were not effective office-based medications for OUD. Once office-based addiction treatment with buprenorphine became available after its Food and Drug Administration approval in 2002, parallel treatment streams emerged with very little crosstalk between them. In one stream, addiction treatment for OUD continues in many behavioral health settings without the use of medications, and in the other stream, office-based treatment has expanded use of medications, but often with poor communication with allies in the behavioral health stream. While all providers may have same goal of offering high quality, evidence-based care to support recovery for patients in recovery, these discrepancies can be difficult to navigate. There is a risk of diversion with buprenorphine, which may dissuade some providers and families from using it. It is often these stories that are highlighted in the lay press, not the success of patients who have achieved sobriety as a result of OAT. Nonetheless, misuse of buprenorphine is far rarer than misuse of other prescription opioids, and when it does occur, many patients who use buprenorphine without a prescription report using it to self-treat withdrawal symptoms [10].

Young adults, however, may be even more likely to experience stigma related to OAT. Treatment providers may believe that more conservative treatment (i.e., without medication) should be tried first. Many providers view OAT as a ‘last resort’ for youth, often waiting until young adults have first relapsed despite non-medication treatment options, or until they have experienced severe adverse consequences of their opioid use (such as overdose). Although this may be well intentioned, this strategy ignores the evidence that OAT leads to improved outcomes including decreased mortality. Young adults also often have family members involved in treatment decisions, as well as state agencies (such as child protection services), multiple treatment providers, teachers, or other trusted adults. If a young adult perceives negative messaging about OAT from any of these trusted adults, it can be difficult for them to navigate differing opinions. This lack of consistency in messaging can be devastating if it leads to blocking the initiation of potentially life-saving treatments. Of note, we have not experienced the same stigma against naltrexone. This may be because it is considered a more appealing option because of its pharmacologic properties of being an antagonist and lack of risk for diversion.

Despite this stigma related to OAT, it is critical to recognize that families, other providers, and those in the community have shared goals. We all want to provide high-quality, evidence-based care to young adults so that they can achieve sobriety, maintain recovery, and reach their full potential. OAT prescribers should understand that the medical field may be mistrusted by some and that others involved in young adults’ recovery may perceive that we are simply writing a prescription to fix a problem. However, waiting until youth have ‘failed’ traditional treatment options or have reached ‘rock bottom’ are outdated approaches to treatment and should be avoided; instead, providers should offer the most effective treatment early in the course of OUD, including use of medications when appropriate.

We propose the following steps to address the stigma associated with OAT for youth:

1. Access to OAT: all patients should be able to access this life-saving treatment. If MOUD cannot be offered at a treatment program for logistical reasons, that program should partner with an outside prescriber.
2. Increased public education: patients and families should have access to the highest-quality evidence and be aware that OAT is an appropriate option for many youth with OUD. This could take the form of public education campaigns highlighting the success stories of those treated with OAT and increased education of health care professionals about the benefits of OAT. It should include increasing contact between people who are doing well in treatment and those who don’t want treatment because of stigma. Families should be explicitly involved in these efforts.
3. Increased education among health professionals: all health care providers, including those who are not prescribers (social workers, recovery coaches, etc.) should receive evidence-based education about OAT.
4. Words matter: although stigmatizing language did not play a direct role in the cases described above, prior work has shown that language matters in how we treat those with substance use disorders [11, 12]. Providers should reframe language around medications, and highlight that opioid agonists buprenorphine and methadone are evidence-based treatments.
5. Media involvement: the media should be held to the same standards and take responsibility to use non-stigmatizing language related to substance use disorders and treatment.

## Abbreviations

MOUD: medication for opioid use disorder
OUD: opioid use disorder
PTSD: post-traumatic stress disorder

## References

[1] Rudd R, Seth P, David F, Scholl L (2016). Increases in drug and opioid-involved overdose deaths—United States, 2010–2015. MMWR Morb Mortal Wkly Rep.

[2] Committee on Substance Use and Prevention (2016). Medication-assisted treatment of adolescents with opioid use disorders. Pediatrics.

[3] Feder KA, Krawczyk N, Saloner B (2017). Medication-assisted treatment for adolescents in specialty treatment for opioid use disorder. J Adolesc Health.

[4] Hadland SE, Wharam J, Schuster MA, Zhang F, Samet JH, Larochelle MR (2017). Trends in receipt of buprenorphine and naltrexone for opioid use disorder among adolescents and young adults, 2001–2014. JAMA Pediatr.

[5] Walley AY (2008). Office-based management of opioid dependence with buprenorphine: clinical practices and barriers. J Gen Intern Med.

[6] Cunningham CO, Kunins HV, Roose RJ, Elam RT, Sohler NL (2007). Barriers to obtaining waivers to prescribe buprenorphine for opioid addiction treatment among HIV physicians. J Gen Intern Med.

[7] Stein BD (2015). Supply of buprenorphine waivered physicians: The influence of state policies. J Subst Abuse Treat.

[8] Wakeman S, Rich JD (2017). Barriers to medications for addiction treatment: how stigma kills. Subst Use Misuse.

[9] Bagley SM, Hadland SE, Carney BL, Saitz R (2017). Addressing stigma in medication treatment of adolescents with opioid use disorder. J Addict Med.

[10] Carroll JJ, Marshall BDL, Rich JD, Green TC (2017). Exposure to fentanyl-contaminated heroin and overdose risk among illicit opioid users in Rhode Island: a mixed methods study. Int J Drug Policy.

[11] Botticelli MP, Koh HK (2016). Changing the language of addiction. JAMA.

[12] Kelly JF, Wakeman SE, Saitz R (2015). Stop talking ‘dirty’: clinicians, language, and quality of care for the leading cause of preventable death in the United States. Am J Med.

